# Modeling of the Human Bone Environment: Mechanical Stimuli Guide Mesenchymal Stem Cell–Extracellular Matrix Interactions

**DOI:** 10.3390/ma14164431

**Published:** 2021-08-07

**Authors:** Ana Rita Pereira, Andreas Lipphaus, Mert Ergin, Sahar Salehi, Dominic Gehweiler, Maximilian Rudert, Jan Hansmann, Marietta Herrmann

**Affiliations:** 1 IZKF Group Tissue Regeneration in Musculoskeletal Diseases, University Hospital Wuerzburg, 97070 Wuerzburg, Germany; r-pereira.klh@uni-wuerzburg.de (A.R.P.); mertergin.de@gmail.com (M.E.); 2 Bernhard-Heine-Centrum for Locomotion Research, University of Wuerzburg, 97074 Wuerzburg, Germany; 3 Biomechanics Research Group, Ruhr-University Bochum, 44801 Bochum, Germany; andreas.lipphaus@rub.de; 4 Department of Biomaterials, Center of Energy Technology und Materials Science (TAO), University of Bayreuth, 95447 Bayreuth, Germany; sahar.salehi@bm.uni-bayreuth.de; 5 AO Research Institute Davos, 7270 Davos, Switzerland; dominic.gehweiler@aofoundation.org; 6 Department of Orthopedic Surgery, Koenig-Ludwig-Haus, University of Wuerzburg, 97074 Wuerzburg, Germany; m-rudert.klh@uni-wuerzburg.de; 7 Fraunhofer Institute for Silicate Research, Translational Center for Regenerative Therapies, 97082 Wuerzburg, Germany; jan.hansmann@isc.fraunhofer.de

**Keywords:** bone tissue engineering, human trabecular bone decellularization, in vitro modeling, shear stress, compressive load, fluid simulation, cell-matrix interaction, mechanotransduction

## Abstract

In bone tissue engineering, the design of in vitro models able to recreate both the chemical composition, the structural architecture, and the overall mechanical environment of the native tissue is still often neglected. In this study, we apply a bioreactor system where human bone-marrow hMSCs are seeded in human femoral head-derived decellularized bone scaffolds and subjected to dynamic culture, i.e., shear stress induced by continuous cell culture medium perfusion at 1.7 mL/min flow rate and compressive stress by 10% uniaxial load at 1 Hz for 1 h per day. In silico modeling revealed that continuous medium flow generates a mean shear stress of 8.5 mPa sensed by hMSCs seeded on 3D bone scaffolds. Experimentally, both dynamic conditions improved cell repopulation within the scaffold and boosted ECM production compared with static controls. Early response of hMSCs to mechanical stimuli comprises evident cell shape changes and stronger integrin-mediated adhesion to the matrix. Stress-induced Col6 and SPP1 gene expression suggests an early hMSC commitment towards osteogenic lineage independent of Runx2 signaling. This study provides a foundation for exploring the early effects of external mechanical stimuli on hMSC behavior in a biologically meaningful in vitro environment, opening new opportunities to study bone development, remodeling, and pathologies.

## 1. Introduction

Bone tissue unveils remarkable mechanical properties and regeneration potential, mainly provided by its particular extracellular matrix (ECM) composition and organization. Bone ECM consists of 30–45% of organic matrix, primarily composed of collagen type I (Col1) assembled in twisted microfibrils [1,2]. These fibrils interact with other collagenous (e.g., type III and V collagen) and non-collagenous proteins (e.g., bone sialoproteins, proteoglycans, osteocalcin, osteopontin (SPP1), etc.), establishing an optimal biochemical and physical environment for bone-resident cells [3]. Simultaneously, Col1 fibrils provide a template for hydroxyapatite crystals to nucleate parallel along their axis and therefore grant mechanical competence to the tissue [4,5].

ECM is, in principle, a very dynamic structure that controls and is controlled by its surrounding environment, adapting its structural arrangement and composition to external stimuli [6,7,8,9]. In fact, bone tissue is mainly subjected to two types of mechanical signals: (1) strain caused by tension or compression triggered by physical activity, and (2) shear stress as a result of interstitial fluid movement through bone lacunae. These deformations are sensed by bone resident cells, namely, osteoblasts and osteocytes, through their ion channels and/or cell membrane receptors (e.g., transmembrane integrins and cadherins) and transduced into intracellular biochemical signals in a process known as mechanotransduction [10,11]. Particularly, fluid shear stress has been shown to induce synthesis of non-collagenous proteins by osteoblasts, and therefore influence mineralization [12,13]. The Wnt/β-Catenin canonical pathway is well established to be activated in response to mechanical stimulation, inducing downstream expression of secretory proteins, such as SPP1, and thereby influencing osteoblastogenesis and bone formation [14,15]. In vitro bioreactor studies have shown that cyclic compression loads prompt osteoblast division, matrix production, and increase levels of alkaline phosphatase (ALP) activity, which result in an improvement of the compressive modulus of the entire structure [16,17]. Furthermore, not only the magnitude and type of stimuli, but also the rate and frequency can influence the quality of new bone formation, and consequently, bone formation efficiency [18].

As such, understanding the detailed biomechanical aspects of bone homeostasis and regeneration is essential for transferring useful knowledge related to the integration of complex stimuli to which cells are subjected, either in physiological or pathological conditions. Generally, there are two possibilities to approach this: (1) experimental methods and (2) computational modeling. Notwithstanding, in silico modeling can provide valuable projections for the design and optimization of experimental strategies [19,20]. However, the complexity of in vivo biochemical and biomechanical features is the primary limitation of these studies, which require extreme processing competencies in order to formulate and solve several finite elements iterations. On the other hand, experimental methods make use of standard models from which representative clinical outcomes are foreseeable; particularly, animal models are currently a keystone of biomedical research. Yet, several limitations are raised when studying human diseases, as experimental animals often fail to recapitulate critical aspects, e.g., the age of patients and the specific human microenvironmental architecture and physiology [21,22,23]. On those grounds, over the last decade, bone tissue engineering strategies have been the focus of the research field as they allow us to recapitulate developmental processes in tridimensional (3D) in vitro settings [9,24]. Different types of materials have been used to engineer bone. Synthetic materials (e.g., polymers, composites, bioceramics, etc.) gained recognition mostly due to their great flexibility, reproducibility, and control over scaffold functionalization, and therefore the possibility to tune their composition, structural, and mechanical properties [25]. However, these materials still represent a very artificial environment to the cells, often exposing problems of biocompatibility and poor osteoinductive properties [26]. Natural-derived polymers (e.g., collagen, fibrin, chitosan, etc.) are constituted of naive ECM and show high biocompatibility but present poor mechanical properties and do not represent the structural organization of the native bone tissue [27]. As a matter of fact, the internal architecture of the scaffold, e.g., particularly the porosity and pore size, is shown to directly affect cell proliferation, signaling, and osteogenic differentiation [28,29]. In recent years, decellularization of tissues or organs has been widely explored since the resulting construct is able to preserve both the biochemistry and the architecture of the native ECM of the respective tissue [30,31]. Specifically for bone decellularization, partial or full demineralization is shown to further enable the exposition of soluble and insoluble osteogenic molecules that otherwise were embedded in the calcified matrix [32]. Therefore, the direct interaction of these molecules with seeded cells reportedly triggers osteogenic differentiation processes and consequently enhances bone formation [33].

In this study, we aim to investigate the early influence of mechanical stimuli on the behavior of bone marrow mesenchymal stem/stromal cells (hMSC) seeded in a physiologically relevant environment. Besides the native chemistry and mechanical properties of the bone environment offered by the decellularized human bone scaffold, a perfusion bioreactor with a uniaxial compression load was used to mimic external forces to which bone cells are subjected in vivo. Finite element analysis of the applied forces did support the reliability of the model.

Finally, the design of multimodal bone tissue models, such as the one developed here, opens new opportunities to validate bone development, remodeling, and pathology studies, as it provides biologically meaningful in vitro systems in which specific experimental parameters can be systematically controlled.

## 2. Materials and Methods

### 2.1. Preparation of Decellularized Bone Scaffolds

Decellularized bone scaffolds were obtained from human trabecular femoral head specimens (permission number: 187/18, University of Wuerzburg ethics committee), as previously described in [34]. Briefly, freshly thawed samples were precisely cut in 3 mm thick slides using an electric diamond band saw (300, Exakt; D64, Walter Messner GmbH, Oststeinbek, Germany) to ensure homogeneous penetration of washing solutions through the complete sample volume. Blood and residual fat material were firstly removed by several cycles of washing in water and a chloroform (288306, Sigma-Aldrich, Steinheim, Germany) and methanol (8388.6, Carl-Roth, Karlsruhe, Germany) mix solution. Further decalcification of bone slices was achieved by incubation for several days in 2.5% ethylenediaminetetraacetic acid (EDTA, E5134, Sigma-Aldrich) in 10 mM Tris-base (T6066, Sigma-Aldrich), from where cylindrical constructs were shaped using a 10 mm biopsy punch. Complete decellularization of bone samples was achieved by enzymatic treatment with 100 Units/mL DNase (DN25, Sigma-Aldrich) and finalized with lyophilization (Martin Christ, Alpha 1–2 LDplus, Osterode am Harz, Germany) for 4 days under a vacuum pressure of 1 mbar. Processed bone scaffolds were stored at −20 °C, and sterilization with 70% ethanol was always performed freshly the day before cell seeding.

### 2.2. Elastic Modulus Measurements

A TA ElectroForce^®^ 5500/BOSE device (New Castle, PA, USA) equipped with a 200 N load cell in an unconfined compression test setup was used to assess the elastic modulus of scaffolds. Both native and decellularized cylindrical samples (3 mm height and 10 mm diameter; 3 technical replicates from 5 donors) were mounted in the setup and exposed to a compression load resulting in a total displacement of 13% (=−0.39 mm) at a rate of 0.001 mm/s or 0.005 mm/s, respectively. WinTest7^®^ (version 7.2) software continuously recorded the resulting force (Newton) applied on scaffolds during testing. Elastic modulus results were obtained by linear regression of strain versus stress values for each sample.

### 2.3. Pore Size Measurements

Decellularized bone scaffolds from five donors were scanned using micro computed tomography (microCT, vivaCT40, SCANCO Medical AG, Brüttisellen, Switzerland), with an x-ray energy of 70 kV and a 0.114 mA tube current with 200 ms integration time and reconstructed with an isotropic voxel size of 0.0105 mm. Image data were exported to ImageJ (version 1.53c) [35]. Data were collected by measuring the Ferret diameter of 50 pores randomly distributed through the full volume of each sample using ROI manual selection and the Analyze menu.

### 2.4. Dynamic Bioreactor

A custom-made bioreactor system, previously described in [36], was designed to mimic the mechanical environment to which bone tissue is subjected in vivo, where both shear stress and cyclic compression can be applied to the cell-seeded scaffolds. Briefly, the system can be separated into three compartments—(1) the bioreactor cartridge, where the cell-seeded scaffolds are harbored inside a 10.4 mm diameter silicone housing, which allows gas exchange while avoiding fluid extravasation; (2) a computer-controllable peristaltic pump that ensures the continuous fluid flow of the cell culture medium through a unidirectional closed-circuit centered between the bioreactor-cartridge and the medium reservoir; and (3) an uniaxial loading unit directly connected with the bioreactor cartridge, where frequency and magnitude of the piston displacement can be tailored. The bioreactor was maintained in aseptic conditions in a controlled chamber at 37 °C and with 5% CO_2_.

### 2.5. Computational Fluid Dynamic

Two computational finite element models were established to calculate wall shear stress, pressure, and velocity in the bioreactor system and the decellularized bone scaffold. First, a model of the full system based on CAD data was set up by importing the geometry to ANSYS Fluent (version 19.2). The volume model was meshed with 195138 nodes. Liquid water with a dynamic viscosity of 0.6913 mPa·s in accordance with a temperature of 37 °C was set as the material model for the fluid. The inlet was modeled as velocity inlet with a velocity of 1.7 mL/min and the outlet as the outflow, as environmental condition atmospheric pressure was used. The scaffold geometry was simplified as a porous medium with a porosity of 90%. To access permeability, the differential pressure of three bone scaffolds of different donors was experimentally measured, and the permeability was calculated according to Darcy’s law. Contact areas between the solid and fluid phases were modeled as interfaces. After initialization using the hybrid initialization method, the model was solved with five iterations. A second detailed model of the bone scaffold was established based on microCT data of decellularized bone scaffolds. Briefly, images of microCT data and chamber geometry were imported into ScanIP (Simpleware, version 14) for segmentation and meshing. After adjusting for size and application of the median filter, segmentations with two different thresholds were combined using a Booleans operation and dilate and close-Filter, as well as Gaussian smoothing. Flood fill was used on segmented masks to remove floating islands. After inlet, outlet, and walls were defined as boundary conditions, the model was meshed using the +FEGrid meshing algorithm as a msh-file containing 36212059 nodes and imported to Fluent (Version 19.2). Boundaries and material properties were set accordingly to the first model.

### 2.6. Mesenchymal Stem/Stromal Cells Isolation and Loading Protocol

hMSCs were isolated from human bone marrow from acetabular reaming in patients undergoing hip arthroplasty surgery after obtaining informed consent of the patient and ethical approval (187/18).

Briefly, mononuclear cells were collected from bone marrow aspirates by a Ficoll (Histopaque-1077, Sigma-Aldrich) density gradient centrifugation and repeatedly washed. The cell count was determined, and cells were cultured for adhesion selection of hMSCs. Cells were further expanded with basal medium (DMEM/F-12 GlutaMAX, Gibco, Bleiswijk, Netherlands) supplemented with 10% fetal bovine serum (FCS, Bio&Sell, Feucht, Germany), 1% Pen/Strep (100 U/mL, Gibco), 1% HEPES (Sigma-Aldrich), and 5 ng/mL fibroblast growth factor (FGF, 100-18C, PeproTech, Hamburg, Germany) until passage 4–6 and seeded in decellularized bone scaffolds as previously described in [34]. Briefly, 50 µL containing 1.5 × 10^6^ cells was distributed on top of each scaffold and incubated for 3 h at 37 °C with 5% CO_2_ to allow cell attachment, followed by 21 h static incubation with the supplemented basal medium.

For each dynamic condition, i.e., (1) only perfusion or (2) perfusion plus compression, three scaffolds were stacked in a bioreactor cartridge, and osteogenic differentiation cell culture medium (DMEM low glucose (Gibco) supplemented with 10% FCS, 1% Pen/Strep, 1% HEPES, 50 μg/mL L-Ascorbic acid 2-phosphate (Sigma-Aldrich), 5 mM β-Glycerophosphate disodium salt (Sigma-Aldrich), and 10 nM dexamethasone (Sigma-Aldrich)) was continuously perfused at 1.7 mL/min. Uniaxial compression loading was applied for 1 h per day at a frequency of 1 Hz and amplitude of 10%, whilst perfusion was temporarily halted.

The total differentiation culture duration was either one or seven days. On day four, half of the cell culture medium was renewed. Cells were harvested for further analysis always 6 h after the last loading cycle. For all experiments, constructs in static conditions were implemented as controls.

### 2.7. Viability Assays

To confirm cell viability and scaffold integration, cell metabolic activity and distribution within the scaffold were assessed both by (1) MTT (3-(4,5-dimethylthiazol-2-yl)-2,5diphenyltetrazolium bromide, 20395.04, Serva Electrophoresis, Heidelerg, Germany) assay and by histological staining of cryostat sections with Hematoxylin and Eosin (H&E).

Briefly, after 7 days of differentiation culture, each scaffold was cut in the middle and incubated at 37 °C for 3 h in 10% MTT (5 mg/mL) solution in a cell culture medium. Pictures from the top and the cross-section view of the entire scaffold were acquired using a stereomicroscope (Zeiss, Discovery V20, Oberkochen, Germany). Blank samples without cells were used as controls.

In parallel, samples were fixed and snap-frozen in Tissue-Tek^®^ O.C.T.™ Compound (4583, Sakura Finetek, Hartenstein, Wuerzburg, Germany), from where 8 µm-thick cryosections were collected and stained with H&E. Scaffold integration and cell proliferation within each sample was examined under a light microscope (Leica, DMi8, Wetzlar, Germany).

Representative images are shown from three individual experiments.

### 2.8. Immunofluorescence Analysis

Immunofluorescence analysis of 7-day samples was performed to visualize the presence and location of either native or newly formed ECM proteins. Briefly, the cryosections were permeabilized with 0.1% (*v*/*v*) Triton-X (3051.3, Carl-Roth) for 30 min at RT and blocked with 1% (*w*/*v*) Bovine Serum Albumin (BSA, A1391, AppliChem, Darmstadt, Germany) for 1 h prior to treatment with primary antibodies (Collagen-type1, anti-Col1, dilution 1:300, ab34710, abcam, Cambridge, UK; Osteopontin, anti-SPP1, dilution 1:500, HPA027541, Sigma-Aldrich) overnight at 4 °C. After washing, samples were incubated with secondary antibody (goat anti-rabbit IgG conjugated with Alexa Fluor^®^ 594, ab150080, abcam) for 2 h at RT. Finally, samples were embedded in Vectashield^®^-containing DAPI (H-1200, Vector Laboratories, Biozol, Eching, Germany) for nuclei staining. In addition, Phalloidin-iFluor 488 (ab176753, abcam) was added during primary antibody incubation for cell cytoskeleton visualization.

Antibody specificity control was performed by incubating the samples in 1% BSA without the first antibody, i.e., anti-Col1 and anti-SPP1, respectively, followed by standard incubation with secondary antibody and detection reagents.

### 2.9. Scanning Electron Microscopy and Energy Dispersive X-ray Spectroscopy

Scaffold structure and hMSC morphology were evaluated for each culture protocol after 30 h post differentiation by scanning electron microscopy (SEM) (Thermo Fischer Scientific, FEI Apero VS, Darmstadt, Germany). The samples were fixed in 4% formaldehyde, dehydrated in a serial dilution of ethanol, dried in tert-butanol, and immediately freeze-dried. Prior to imaging, all samples were sputter-coated (Leica, EM ACE600) with a 2 nm film of platinum to ensure conductivity of the sample’s surface. Images were taken at an accelerating voltage equal to 1.5–3 kV and a magnification of ×200 and ×5000. Cellular details were artificially colored on magnified images using Photoshop^®^ CS6 (Adobe, v13.0.1) for visualization purposes.

In addition, a dispersive energy X-ray (EDS) detector (Carl Zeiss, Gemini Sigma 300 VP, Oberkochen, Germany) operating at 10 KeV was used to determine the surface atomic composition of the decellularized bone scaffolds. Three random areas of interest were evaluated for each sample.

### 2.10. Gene Transcription Analysis

To analyze the early gene response of hMSC to mechanical stimuli, two scaffolds for each condition were harvested in Trizol (Sigma), and cells were lysed for 5 min at 50 Hz (TissueLyser LT, Quiagen, Hilden, Germany). RNA was isolated by 1-bromo-3-chloropropane (B9673, Sigma-Aldrich) phase separation followed by column separation according to the manufacturer’s instructions (NucleoSpin RNA, Macherey-Nagel, Dueren, Germany). cDNA was synthesized by reverse transcription (Promega, Walldorf, Germany) from 1 µg of RNA.

Real-time Polymerase chain reaction (PCR) was performed by CFX96 Real-Time System (Bio-Rad). Primers for genes targeting either mechanosensory functions—fos proto-oncogen (cFos), prostaglandin-endoperoxide synthase 2 (Cox2), integrin subunit beta 5 (ITGb5), osteopontin (SPP1), bone morphogenetic protein 2 (BMP-2)—or osteochondral early differentiation—collagen type-VI (Col6), runt-related transcription factor 2 (Runx2), SRY-Box Transcription Factor 9 (Sox9)—were designed in the Primer Blast tool from NBCI and purchased from Biomers.net (Ulm, Germany). Primers sequence and NCBI reference numbers appear in Supplementary Table S1.

Expression of target genes was normalized with beta-2-microglobulin (B2M, NM_004048.2) as the reference gene, and results displayed as relative values (10,000 × 2^−^^ΔCt^).

### 2.11. Statistical Analysis

Qualitative data were analyzed using Graphpad Prism software (version 9.1) and presented as the mean ± standard error of the mean. Statistical significance was investigated using the Kruskal–Wallis method followed by Dunn’s multiple comparisons test. Statistical significance was set at *p* ≤ 0.05.

## 3. Results

### 3.1. Scaffold Structure Characterization

**Figure 1:**
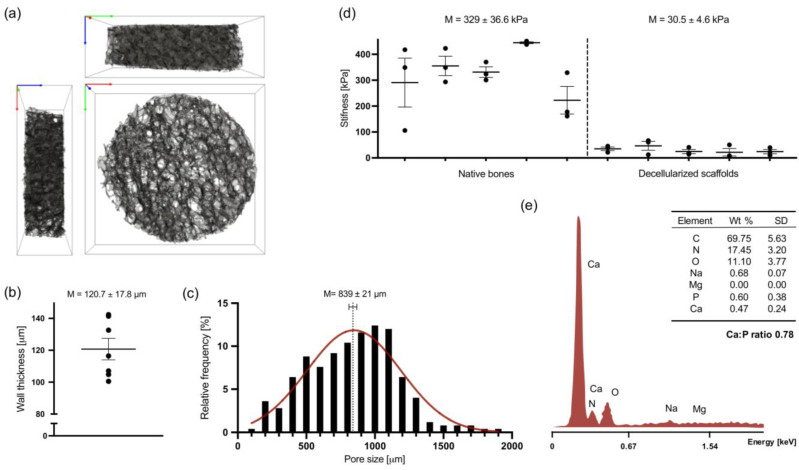
Structural characterization of human femoral head-derived decellularized bone scaffolds. (**a**) A 3D volume rendering obtained from mesh segmentation (10^6^ tetrahedra elements) of microCT scan of a representative scaffold. Box dimension: 10 × 10 × 3 mm. Axis: x—green, y—red, z—blue. (**b**) Wall thickness, or trabeculae thickness (Tb.Th), calculated from the total volume of interest (VOI) determined on Simpleware^TM^ ScanIP respectively for each segmented mesh sample (n = 7). (**c**) Histogram of the relative frequency of pore size diameter (n = 5). Data assume a Gaussian distribution shown in red. (**d**) Stiffness of native and decellularized constructs, shown as means of elastic modulus obtained by mechanical compression testing (n = 5). The same shape and size were used for measurements of both native and decellularized samples. (**e**) Representative EDS spectra of decellularized scaffolds surface and respective quantification of atomic element weight percentage (wt%, n = 2). C—carbon, N—nitrogen, O—oxygen, Na—sodium, Mg—magnesium, P—phosphorus, and Ca—calcium.

The microstructure of decellularized bone scaffolds was evaluated by microCT reconstruction (Figure 1a). Three-dimensional porous scaffolds with highly interconnected anisotropically distributed pores were obtained from a combination of chemical, enzymatic and physical decellularization of human femoral head-derived trabecular bone.

Analysis of trabecular thickness (Figure 1b) and pore size distribution (Figure 1c) confirmed a preserved native bone tissue architecture with an overall trabeculae thickness average of 120.7 ± 17.8 µm and a wide Gaussian pore size distribution ranging from 100 to 2000 µm.

The stiffness of both native and decellularized constructs was determined by mechanical compression of 10% of the total scaffold length, i.e., approximately 0.3 mm (Figure 1d). Despite high both inter-and intra-donor variance, the elastic modulus of decellularized scaffolds (30.5 ± 4.6 kPa) evidently decreased about tenfold in comparison with native trabecular constructs (329 ± 36.6 kPa) due to the EDTA-induced decalcification occasioned by the decellularization protocol. Yet, EDS spectra obtained from decellularized bone scaffolds surface (Figure 1e) shows the remaining presence of bone minerals, such as calcium and phosphorus, 0.47 and 0.6 weight %, respectively.

### 3.2. Computational Modeling

**Figure 2:**
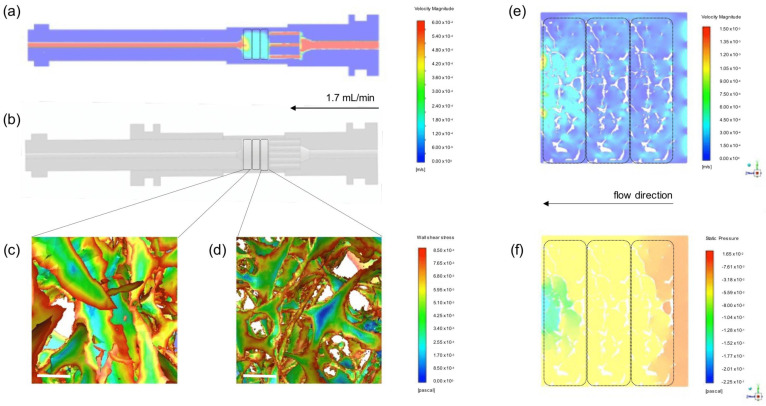
Computational fluid simulation. (**a**) Velocity profile of the simplified CAD model. Perfusion direction from the right (inlet) to the left (outlet). (**b**) A 3D rendering model of bioreactor parts visualized on AutoDesk Inventor software. (**c**,**d**) Close-up view of wall shear stress calculated in the microCT model for the scaffolds nearest the outlet and inlet, respectively. Scale-bar: 200 µm. (**e**) Cross-section of the velocity profile in the fluid phase from microCT model surrounding the bone scaffolds. (**f**) Cross-section of the pressure profile in the fluid phase from microCT model surrounding the bone scaffolds. In overview figures, the region where scaffolds are positioned is highlighted with traced line.

In order to estimate mechanical conditions sensed by hMSCs in this specific complex in vitro system, finite element models of both (1) a simplified CAD model of the bioreactor and (2) a complex microCT model of the bone scaffold structure were established. For the porous media, permeability k was calculated taking into consideration the fluid flow *Q* = 1.7 mL/min, dynamic viscosity η = 0.6913 mPa·s (assumed for water at 37 °C), length of the bone scaffolds *L* = 9 mm, the cross-sectional area of the bioreactor cartridge *A* = 78.5 mm^2^ and experimentally measured differential pressure Δ*p* using Darcy´s law $k=\frac{Q\;\mu \;L}{A\;\Delta p},$ resulting in a mean permeability of 6.64 × 10^−12^ ± 1.22 × 10^−12^ m^2^. Using this value for the simplified CAD model, the velocity in the bioreactor cartridge was calculated as 0.16 mm/s. Please note that the velocity in the pipes is higher than in the bioreactor cartridge because of the different diameters of the tubing (Figure 2a). The fluid-induced wall shear stress on the scaffold in the microCT model was on average 8.5 mPa. Wall shear stress did not markedly differ between the first (Figure 2d) and third scaffold (Figure 2c). However, the highest values of wall shear stress were observed at the edges of the bone scaffold, while larger internal areas further away from the pores showed lower to zero wall shear stress values. The mean velocity in the fluid phase was calculated for the microCT model as 0.166 mm/s (Figure 2e), matching the result of the simplified CAD model, and the mean pressure as 40 mPa (Figure 2f). Notably, velocity peaked near the inlet and outlet pipes, while pressure dropped from the highest value near the inlet to the lowest values at the outlet in relation to the atmospheric pressure.

### 3.3. In Vitro Studies

#### 3.3.1. hMSC—Scaffold Integration in the Static and Dynamic Culture

**Figure 3:**
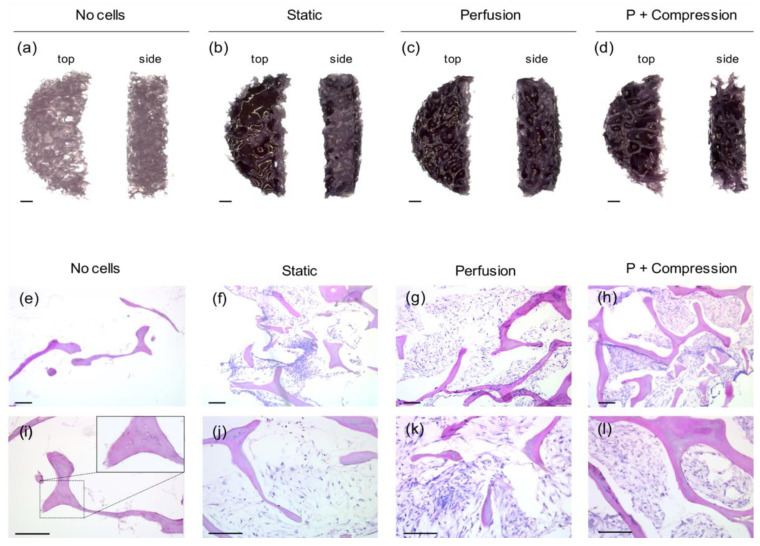
hMSC viability and scaffold integration after 7 days in osteogenic culture in different regiments. (**a**–**d**) Representative images of cell viability MTT staining of the top (left) and cross-section (right) view of scaffolds acquired by RGB-stereomicroscopy. (Scale-bar: 100 µm, n = 3). (**e**–**l**) Representative images of H&E staining from 8 µm-thick scaffold cryosections. Successful hMSC integration within the decellularized bone scaffold (strong pink color) is observed for all cell-laden conditions (purple color), while ECM deposition (light pink color) is rather visible in both perfusion (**k**) and perfusion + compression (**l**) conditions. A close-up zoom image (right upper corner) of non-cellular scaffolds (**i**) shows empty lacunae without the presence of osteocytes. (Scale-bar: 200 µm, n = 3).

The impact of different cell culture regiments, i.e., static culture, flow perfusion, and combination with compressive loading, on hMSCs was initially studied after 7 days of culture. hMSC viability and distribution through the scaffold volume were evaluated by MTT staining (Figure 3a–d). Strong and consistent positive staining was observed for all conditions, indicating that the decellularized human bone scaffolds indeed provide a base for cell attachment and proliferation, promoting cell viability over time. Particularly better homogeneous distribution is visible in 3D scaffold cross-sections where perfusion is present, i.e., both perfusion only (Figure 3c) and perfusion + compression (Figure 3d) regiments.

To support this observation, histological staining of scaffolds’ cryosections (Figure 3e–l) further shows a superior scaffold integration of hMSC in both dynamic conditions (Figure 3h,l) compared with static culture images (Figure 3f). Likewise, major cell growth is apparent only in dynamic conditions; there, hMSC are tidily densely packed around the high porous scaffold structure, and ECM deposition is observed at higher magnifications (Figure 3k,l).

Sections of non-cellular scaffolds (Figure 3e,i) show the presence of canaliculi and empty lacunae, confirming once more the maintenance of trabeculae native structures and the efficiency of the decellularization protocol.

**Figure 4:**
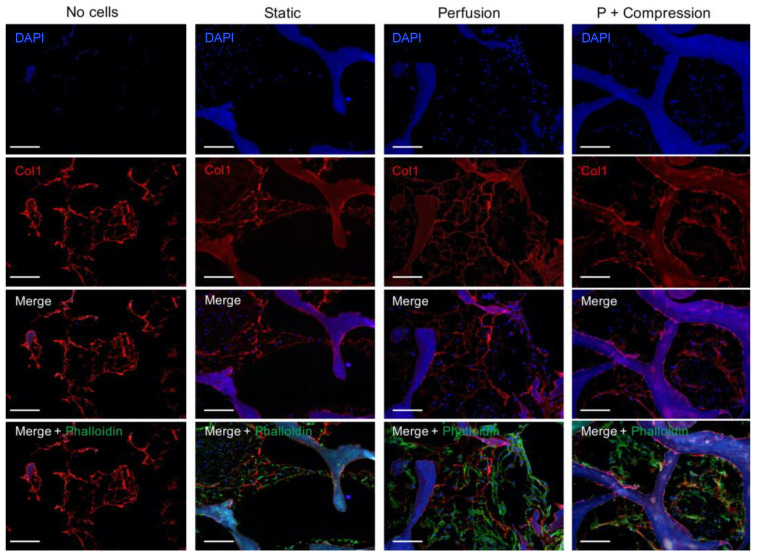
Immunofluorescence analysis of Col1 in acellular and cellular constructs in different culture conditions. Individual channels of nuclei (DAPI, blue) staining and Col1 (red) are shown in the first two rows, followed by the corresponding overlay images, also including cell cytoskeleton staining (Phalloidin, green). Due to an intrinsic auto-fluorescence of decellularized bone scaffolds for most of the common fluorescence channels, e.g., DAPI and FITC, the brightness of green-channel images was occasionally altered with Photoshop for visualization purposes. The biological interpretation of the images was not distorted. (Scale bar 200 µm, n = 3).

**Figure 5:**
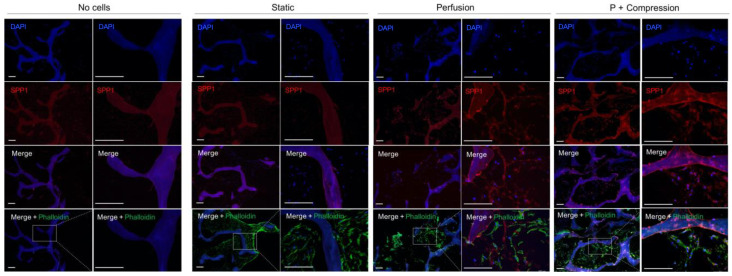
Immunofluorescence analysis of SPP1 in acellular and cellular constructs in different culture conditions. Individual channels of nuclei (DAPI, blue) staining and Col1 (red) are shown in the first two rows, followed by the corresponding overlay images, also including cell cytoskeleton staining (Phalloidin, green). Specificity control without the anti-SPP1 antibody is shown in Supplementary Figure S1. For each condition, a representative higher magnification picture is shown on the left, and a detailed high magnification from the field of interest is shown on the right. (Scale bar 200 µm, n = 3). Due to an intrinsic auto-fluorescence of decellularized bone scaffolds for most of the common fluorescence channels, e.g., DAPI and FITC, the brightness of green-channel images was occasionally altered with Photoshop for visualization purposes. The biological interpretation of the images was not distorted. (Scale bar 200 µm, n = 3).

hMSC integration within the scaffold, particularly cellular interaction with ECM bone proteins, was further investigated by immunofluorescence staining of Col1 (Figure 4) and SPP1 (Figure 5).

An organized collagen fibrilar network is observed for all samples, including in the non-seeded control scaffold, validating the biochemical and structural preservation of collagen fibers as the most dominant organic ECM component in native bone tissue. Interestingly, a specific co-localization between collagen fibers and the presence of hMSC is observed, indicating the close contact between the cells and collagen fibers as an anchoring structure for cell attachment and proliferation. Confirming the above results, a higher cell density for both dynamic conditions is observed, where the collagen fiber’s structure seems to be broadly arranged in space, in contrast with the static environment where the high cell density seems to shrink the soft matrix fibers.

Distinctively, an intrinsic SPP1 expression is mainly detected within the trabeculare structures of decellularized bone scaffolds in all samples. Note that the positive signal is only attributed to SPP1-specific binding since that secondary antibody control (Figure S1) shows no detectable autofluorescence signal. Locally nascent SPP1 deposition is further observed in the pericellular matrix space only in mechanically stimulated conditions, i.e., perfusion only and perfusion and compression.

ALP expression (data not shown) was also investigated for all conditions showing common positive staining, yet no obvious differences were detected between the different groups for this time point.

#### 3.3.2. hMSC Early Response to Mechanical Stimuli

**Figure 6:**
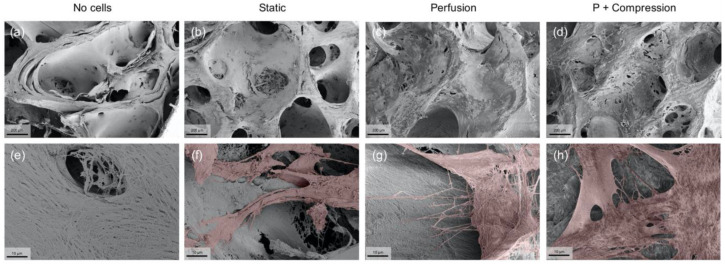
Representative SEM images of hMSC’s early morphological response to mechanical stimuli. (**a**–**d**) 200× low magnification images confirm once more the presence of a highly porous structure, where hMSCs attach physically to the scaffold walls. (Scale bar 200 µm). (**e**–**h**) 5000× high magnification images show single cell interactions with the ECM through cytoplasm extensions, such as filopodia, present in samples subjected to dynamic culture. For visualization purposes only, cell surface areas were artificially colored in red with Photoshop. (Scale bar 10 µm).

**Figure 7:**
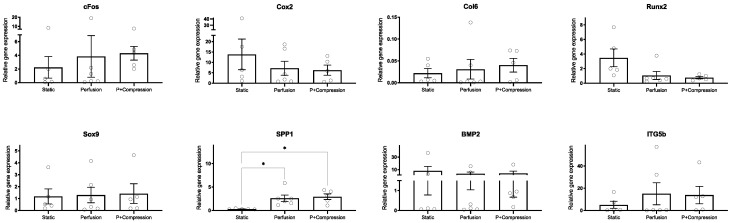
Gene expression analysis of hMSC’s early response to mechanical stimuli. Expression of targeted genes involved in mechanosensory functions (cFos, Cox2, ITGb5, SPP1, BMP-2) unveil a general upregulation in dynamic conditions compared with static, whereas osteochondral early differentiation markers (Col6, Runx2, Sox9) expression seems to be inconclusive for this early time point. Expression of target genes was normalized with B2M as reference gene, and results displayed as relative values (10000 × 2^−^^ΔCt^). Statistical analysis using student Kruskal–Wallis one-way method followed by Dunn’s multiple comparisons tests (* *p* < 0.05, n = 5–6).

Considering the successful results of hMSC scaffold integration and bone-ECM protein accumulation, particularly under mechanical stress induced conditions, the early response of hMSC to mechanical stimuli was further investigated. Both hMSC morphology (Figure 6) and gene expression analysis (Figure 7) were assessed after only one cycle of loading, i.e., the total time of incubation was adjusted to 30 h.

Promptly, low magnification SEM images reveal a less-efficient hMSC integration in static (Figure 6b), compared with highly efficient cell dispersion in both dynamic conditions (Figure 6c,d). These results confirm and support the previous experiments, uncovering the early determination of superior homogeneous cell distribution inflicted by dynamic conditions.

In high magnification images, it is possible to observe further the complex organic structure of the acellular scaffold (Figure 6e), mainly composed of collagen network fibers naturally present in the trabecular bone, as shown by immunofluorescence assays. Very interestingly, single hMSCs in both dynamic conditions (Figure 6g,h) seem to exhibit several filopodia extensions granting the cells to interact with the ECM environment actively, whereas in static conditions (Figure 6f), hMSC seems to display a steadier morphology, where the observed cell extrusions are smaller and rather orientated towards cell–cell than cell–ECM integrations.

Regarding the gene expression analysis (Figure 7), large donor-specific variations were observed due to the primary origin of hMSCs. Donor-to-donor differences are widely reported in the literature in various contexts, recognized to be a reflection of not only the age or overall health of the donor, but also of the diversity of environments from which hMSCs may be isolated [37,38].

hMSCs cultivated in both dynamic conditions showed, as expected, a trend of upregulation for mechanosensitive factors (cFos, ITGb5, SPP1). Despite this trend, statistical analysis indicated no significance (*p* > 0.05) for most of the genes, except for SPP1. Cox2, a very early mechanical responsive gene, showed an opposite trend. BMP-2 expression, despite specific donor variation, does not seem to be significantly affected by mechanical stimulation in our experiments.

Considering markers related to osteochondral differentiation (Col6, Runx2, Sox9), no clear lineage commitment could be observed. Col6 appears to be upregulated in dynamic conditions, particularly when compressive loading is imposed, indicating an augmented differentiation into osteogenic lineage of hMSCs; in contrast, Runx2 was downregulated upon dynamic stimulation, while Sox9 expression was not altered.

## 4. Discussion

In vivo, hMSCs reside in specialized niches that are known for regulating stem cell fate throughout their life span [39,40]. In this work, we established a human decellularized bone scaffold as a model to mimic the native human bone extracellular matrix microenvironment. For the first time, we combined the naïve biochemical and architectural properties retained in these scaffolds with a dynamic culture system, reflecting the physiological mechanical forces to which native bone is subjected in vivo. Our data suggest that hMSCs sense and dynamically adapt to their environment mainly through cell–ECM interactions.

Decellularization techniques have been used in the bone tissue engineering field with the purpose of creating an immunogenic free material while preserving the native tissue structure and its innate osteoinductive qualities [30,31]. Rodriguez et al. [41] identified by mass spectrometry the maintenance of structural ECM proteins (e.g., Col1, Col4, Col6, etc.) present in human bone-derived demineralized fibers, in addition to several growth factors (e.g., BMP-2, BMP-4, BMP-7) and numerous other proteins supporting a variety of intra- and extracellular signaling pathways (e.g., fibronectin, fibrinogen, vitronectin, and laminin). Furthermore, it has been reported that partially or fully demineralized bone can provide not only osteoinductive factors, but also superior mechanical, biochemical, and architectural properties supporting scaffold functionality into physiological conditions [42,43]. We have previously developed a protocol based on physical, chemical, and enzymatic methods to consistently achieve human-femoral head-derived decellularized bone scaffolds [34]. The effective removal of all cellular components was previously confirmed, and the cell seeding protocol was optimized to ensure sustained cell viability. Here, we show the suitability of these scaffolds to investigate the response of hMSCs to mechanical stimuli in a naïve ECM environment.

Scaffolds’ ultra-topography, particularly their trabeculae structure and pore size, has been shown to have a major influence on hMSCs proliferation and osteogenic commitment [44,45]. Interestingly, Smith et al. [42] concluded that decellularized scaffolds derived from elderly bone donors with Tb.Th values comparable with the ones obtained in the present study (average of 120.7 µm) showed improved osteoinductive capacity with higher osteogenic gene expression and ALP activity [46]. Due to the propitious combination of abundant surface area for cell attachment and high distribution of large and interconnected pores (average of 839 µm,) cell penetration, growth, and exchange of oxygen and nutrients are wholly provided [47,48,49].

Not only is the physical arrangement of the environment the main constraint to resembling the in vivo niche, but the stiffness of the matrix is likewise widely recognized and accounted for favoring cell–ECM mechanosignaling [50]. However, as a result of the partial decalcification protocol, the stiffness of the native trabecular bones exhibits much higher values (average of 329 kPa) compared with decellularized bone scaffolds (average of 30.5 kPa); the stiffness value obtained compares to previous studies. In fact, matrices with an elastic modulus between 25–40 kPa have been demonstrated to favor osteogenic lineage differentiation, associated with distinctively high expression of the osteogenic transcription factor Runx2 [51,52]. An essential element of human bone structure is hydroxyapatite, a mineral form based on Ca and P, conferring rigidity and mechanical competence to the tissue. However, in bone decellularization protocols, partial decalcification is often applied, mainly for handling purposes, but also for improving osteoinductive properties. Urist et al. [32] showed decades ago that the decalcification of native bone is able to not only retain bone morphogenic proteins and growth factors entrapped in the bone matrix, but even to further expose them and therefore facilitate specific cell–ECM interactions [53,54].

Yet, in the present study, a residual presence of both minerals was visible by EDS (Ca/P ratio = 0.78), which is significant to potentially trigger de novo mineral nucleation [55,56,57]. Future experiments will validate these findings.

Computational fluid simulations allow obtaining non-invasively and in high-resolution knowledge about mechanical conditions inside a 3D structure. Shear stress and hydrostatic pressure are assumed to be the main mechanical stimuli sensed by hMSCs [58,59,60], and finite element analysis can calculate their values even for extremely complex geometries. Models simplifying the bone scaffold to porous media as well as based on microCT scans have been reported using a broad range of perfusion velocity, porosity, and permeability. Shear stress caused by fluid perfusion is in fact widely recognized in the literature as a trigger of hMSC pro-osteogenic commitment (reviewed in [61]). Particularly, Melke et al. [62] concluded that a wall shear stress of 0.5–10 mPa promotes mineralization. Here, we present experimental data of superior scaffold integration of hMSCs due to dynamic rather than static conditions, while computational simulation revealed a mean wall shear stress of 8.5 mPa. Previously, simulations on a similar bioreactor setup showed that intermittent shear stress ranging from 0 to 13.35 mPa was able to induce osteogenic differentiation of rat-derived bone marrow stem cells seeded on a synthetic copolymer scaffold with a comparable porosity to ours, but with higher permeability, in the absence of any chemical stimuli [63]. Notably, our results show that mechanical conditions vary slightly depending on the position considered, i.e., velocity is highest near the inlet and outlet and more homogenous in the middle, while pressure showed a gradient between the inlet and outlet. The potential of microCT scanning is widely recognized for studying complex structures and fine details [64,65], e.g., inter-pore walls and/or pore size wide distribution, which are not taken in consideration in simplified models and can cause bias in the final analysis. Here, map visualization of wall shear stress in the microCT model reveals no distinct differences between the position in the bioreactor cartridge; rather, the heterogeneous complex geometry of the trabecular bone directs the shear stress sensed locally by the cells.

The here-developed human femoral head-derived decellularized bone scaffolds were consistently shown to provide hMSCs a valuable environment able to boost cell viability and ECM production, particularly in dynamic conditions. The presence of continuous perfusion has been shown to improve cell distribution throughout the scaffold, avoiding a preferential accumulation of cells on the edges as observed in static conditions. This is in line with previous studies where perfusion bioreactors and loading systems have been shown to not only provide appropriate oxygen and nutrient supply, but also to have a direct accelerating effect on hMSC matrix production quality and quantity [66,67,68].

Furthermore, dynamic conditions also seem to influence cell-ECM interactions; in particular, mechanical stimulation seems to prevent the Col1 network shrinkage frequently reported in the literature to be provoked by high cell densities in static conditions [69,70]. Therefore, the wide Col1 network observed in dynamic conditions provides hMSCs with a higher surface area throughout the trabecular pores for adhesion and migration with abundant oxygen and nutrients access. Besides the Col1 scaffolding function as a major structural protein in the bone ECM, several studies have shown that ECM proteins, including collagen and non-collagen proteins, have a direct effect on both osteoconduction and osteoinduction [3,71]. Elango et al. [72] showed that Col1 fibrils modulate osteogenesis by binding to integrins of progenitor cells, trigging the differentiation cascade through MAPK-Runx2 activation. Likewise, due to the dual role of SPP1 as a protein-containing pro-adhesive tripeptide motif -RGD [73,74], as well as ECM calcium sequestering competence [75], native expression of SPP1 also plays a fundamental role in the dynamics of bone ECM. In fact, several studies have previously recognized an early SPP1 mRNA and protein expression in response to mechanical stress, elucidating its role in bone remodeling by affecting both osteoclastogenesis and osteoblastogenesis [76,77,78,79]. Consistently, we detected intrinsic SPP1 accumulation within the trabecular structure of all samples after 7 days of differentiation, yet locally nascent deposition in the pericellular matrix space was only clearly observed in hMSCs subjected to dynamic culture, validating the SPP1 role in response to mechanical stimuli.

In fact, an early response of hMSCs to mechanical conditions was observed even after only 1 day of stimulation. One relevant factor for hMSCs’ mechanotransduction response is the exposure time to mechanical signals [80]. Our results show that dynamic culture has a pivotal role in determining the initial spreading of cells into the highly porous 3D scaffold. Furthermore, the combined effect of physical cues of the decellularized bone scaffold ultrastructure and the applied mechanical stimuli are able to control single-cell morphology. Indeed, specific micro- or nanoscale patterns capable of guiding early hMSCs differentiation commitment simply by confining cell shape are often reported in the literature [81,82,83]. In this study, a more spread morphology and the presence of cytoplasmic extensions, known as filopodia, able to sense and interact with the environment [84,85] were observed precisely in hMSCs exposed to dynamic conditions, which further corroborates the functionality and relevance of the human bone in vitro model achieved in this study. Supporting this observation, gene expression of hMSCs cultured in dynamic settings revealed signs of a stress-induced early osteogenic commitment, which has to be proofed in long-term experiments in the future. We observed a higher expression of cFos in hMSCs subjected to dynamic conditions, particularly when perfusion was combined with compression, while Cox2 transient expression after stimuli was probably outpaced at this timepoint. The expression of cFos and Cox2, genes associated with osteogenic mechanotransduction, is known to be quick and short lived [86,87]. Müller-Deubert et al. [88] reported a transient Cox2 mRNA expression in hMSCs for 2 h after stress with a peak of expression at 30 min. Nevertheless, both cFos and Cox2 mechanosensitive genes are described to follow a coordinated expression pattern attributed to early osteogenic but not chondrogenic differentiation [89,90].

In addition, we observed a remarkable upregulation of SPP1 for both dynamic conditions compared with static culture, followed by an increased protein accumulation seen in histology. As previously discussed, SPP1 is an abundant non-collagenous bone matrix protein with multifaceted functions involving cell interactions and ECM modulation [91,92]. Corroborating our findings, SPP1 has been frequently described as a mechanoresponsive target [76], and therefore has been shown to be critical for unloading-induced bone remodeling shown in vitro [93] and in vivo [79,94]. Likewise, upregulation of Col6 was detected—Col6 is a bone anabolic ECM protein and a constituent of the basement membrane involved fundamentally in cell adhesion [95,96], yet simultaneously exhibits a stimulatory effect on osteogenesis in vitro [97,98]. Thereby, it is reasonable to assume that hMSCs establish stronger attachments to their environment in response to mechanical stress by increasing Col6 and SPP1 matrix deposition since both proteins contain RGD peptide sequences as well as connection domains to focal adhesion-related proteins. This assumption is further validated by the increased expression of ITG5b in hMSCs subjected to dynamic conditions in our study, which may, at a cellular level, trigger a cascade of downstream osteogenic differentiation in hMSCs [74,99,100,101].

In contrast, no clear expression pattern could be seen for BMP2, due to high donor variance. BMP2 is similarly a recognized target of mechanotransduction [102,103], and its role in bone repair is well established [104,105]. In fact, BMP2 autocrine signaling is known to be required for the downstream transcription of load-induced Runx2 by hMSCs [106,107], which may explain the low expression of Runx2 observed in our results as well. On the other hand, constant expression of Sox9 suggests that possible chondrogenesis commitment induced by mechanical stimuli [108,109] was absent in our model.

Taken together, the novelty of this study lies in the shared resembling of both biochemical and mechanical properties of human bone tissue elements in a simple in vitro model. Therefore, by providing a better understanding of mechanobiological interactions of cells with their environment, we aim to further identify key interactions to efficiently direct bone formation in homeostasis or pathologic scenarios.

## 5. Conclusions

Bone tissue-engineered constructs often fail to recapitulate either the chemistry, the ultrastructural physical cues, or the external mechanical properties that native bone is exposed to in the human body. In this study, the synergic effects of (1) 3D decellularized human trabecular bone-derived scaffold properties, namely, its trabecular morphology, heterogeneous porosity, and retained osteoinductive factors, and (2) the dynamic culture imposed by perfusion flow and compressive load are shown to be a promising strategy to accurately mimic the complex regiments occurring in vivo.

Here, we first elucidated the structure–function relations in our system by modeling the fluid flow through the highly complex structure interstices of the decellularized bone scaffold by integrating microCT data. Experimentally, we validated the improved effect of dynamic conditions in scaffold integration of hMSCs and reported a boost of ECM production. The early response of hMSCs to mechanical stimuli manifested in evident cell shape changes and stronger integrin-mediated adhesion to the matrix, promoting hMSCs commitment towards osteogenic lineage independently of Runx2 expression.

Although this study has demonstrated favorable improvements towards the synergetic effect of mechanical stimuli in a native human bone environment in vitro, there are still limitations that need to be addressed. Particularly, further long-term osteogenic functional assays would be crucial to accelerate its prospects in the translation of routine scientific practices in the bone engineering field.

## Data Availability

Data not shown can be obtained from the corresponding author upon reasonable request.

## Supplementary Materials

The following are available online at https://www.mdpi.com/article/10.3390/ma14164431/s1, Figure S1: Immunofluorescence secondary antibody control. The absence of detectable signal in the red channel confirms that positive staining is produced from detection of the antigen by the primary antibody (anti-Col1 or anti-SPP1, respectively) and not by the detection system or auto-fluorescence of the sample. Representative images of hMSCs in perfusion + compression condition. (Scale bar 200 µm). Table S1: RT-qPCR primer sequences were generated with the Primer-BLAST tool from NCBI for this study. Gene name, forward and reverse primer sequence, NCBI reference number, and product length (pb: pair of bases) is shown.
