# Supplementary material for: Modeling of the Human Bone Environment: Mechanical Stimuli Guide Mesenchymal Stem Cell–Extracellular Matrix Interactions
Source: Materials (Basel). 2021 Aug 7;14(16):4431. doi: 10.3390/ma14164431 (PMC8398413; doi:10.3390/ma14164431)
Supplement: Supplementary file 1 [file materials-14-04431-s001.zip › materials-1304719-supplementary.pdf]

materials

MDPI

Supplementary Materials

Modeling of the Human Bone Environment: Mechanical Stimuli Guide Mesenchymal Stem Cell–Extracellular Matrix Interactions

Ana Rita Pereira [1,2], Andreas Lipphaus [3], Mert Ergin [1,4], Sahar Salehi [4], Dominic Gehweiler [5], Maximilian Rudert [6], Jan Hansmann [7] and Marietta Herrmann [1,2,*]

[1] IZKF Group Tissue Regeneration in Musculoskeletal Diseases, University Hospital Wuerzburg, 97070 Wuerzburg, Germany; r-pereira.klh@uni-wuerzburg.de (A.R.P.); mertergin.de@gmail.com (M.E.)
[2] Bernhard-Heine-Centrum for Locomotion Research, University of Wuerzburg, 97074 Wuerzburg, Germany
[3] Biomechanics Research Group, Ruhr-University Bochum, 44801 Bochum, Germany; andreas.lipphaus@rub.de
[4] Department of Biomaterials, Center of Energy Technology und Materials Science (TAO), University of Bayreuth, 95447 Bayreuth, Germany; sahar.salehi@bm.uni-bayreuth.de
[5] AO Research Institute Davos, 7270 Davos, Switzerland; dominic.gehweiler@aofoundation.org
[6] Department of Orthopedic Surgery, Koenig-Ludwig-Haus, University of Wuerzburg, 97074 Wuerzburg, Germany; m-rudert.klh@uni-wuerzburg.de
[7] Fraunhofer Institute for Silicate Research, Translational Center for Regenerative Therapies, 97082 Wuerzburg, Germany; jan.hansmann@isc.fraunhofer.de
* Correspondence: m-herrmann.klh@uni-wuerzburg.de

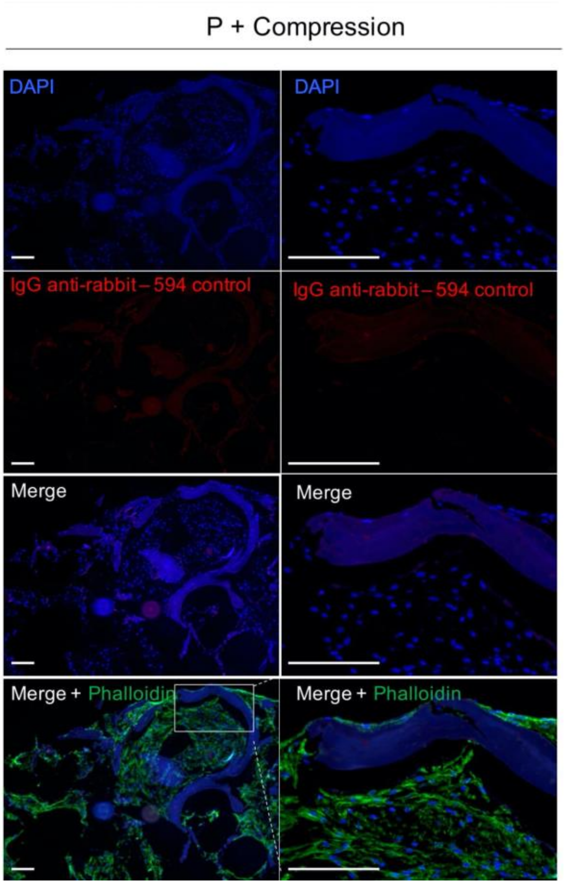

Figure S1. Immunofluorescence secondary antibody control. The absence of detectable signal in the red channel confirms that positive staining is produced from detection of the antigen by the primary antibody (anti-Col1 or anti-SPP1, respectively) and not by the detection system or auto-fluorescence of the sample. Representing images of hMSC in perfusion + compression condition. (Scale bar 200 μm).

Table S1. RT-qPCR primer sequences were generated with Primer-BLAST tool from NCBI for this study. Gene name, forward and reverse primer sequence, NCBI reference number, and product length (pb: pair of bases) is shown.

Gene	Forward sequence 5′-3′	Reverse sequence 5′-3′	NCBI number	Product length (pb)
BMP-2	GGAAC-GGACATTCGGTCCTT	CACCATGGTCGAC-CTTTAGGA	NM_001200.4	127
cFos	GGGGCAAGGTGGAACAG-TTA	AGTTGGTCTGTCTCCGCTTG	NM_005252.4	139
Cox2	CAAATTGCTGG-CAGGGTTGC	AGGGCTTCAGCATAAAGCGT	NM_000963.4	139
ITGb5	CCGGCTCGCAGGTCTCA	CACCAGGCACATTTTGGGTG	NM_002213.5	87
SPP1	TCTGGGAGGGCTTGGTTGTC	GGTAGTGAGTTTTCCTT-GGTCG	NM_000582.2	124
BMP-2	GGAAC-GGACATTCGGTCCTT	CACCATGGTCGAC-CTTTAGGA	NM_001200.4	127
Col6 a1	TCTCAGATGGCAACTCG-CAG	ACCACGAAGATCTCGATGCC	NM_001848.3	91
Runx2	GAGTGGACGAGGCAA-GAGTT	CTGTCTGTGCCTTCTGGGTT	NM_001024630.3	127
SOX9	GCAGGCCGACTCGCCACAC	GGATTGCCCCGAG-TGCTCGCC	NM_000346.3	73
